# *Diplotaxis* Genus: A Promising Source of Compounds with Nutritional and Biological Properties

**DOI:** 10.3390/molecules29112612

**Published:** 2024-06-01

**Authors:** Sandrine Ressurreição, Lígia Salgueiro, Artur Figueirinha

**Affiliations:** 1 University of Coimbra, Faculty of Pharmacy, 3000-548 Coimbra, Portugal; sandrine@esac.pt (S.R.); ligia@ff.uc.pt (L.S.); 2 Polytechnic of Coimbra, Coimbra Agriculture School, 3045-601 Coimbra, Portugal; 3 Chemical Engineering and Renewable Resources for Sustainability (CERES), Department of Chemical Engineering, University of Coimbra, 3030-790 Coimbra, Portugal; 4 Associated Laboratory for Green Chemistry (LAQV) of the Network of Chemistry and Technology (REQUIMTE), University of Coimbra, 3000-548 Coimbra, Portugal

**Keywords:** *Diplotaxis* genus, Brassicaceae, nutritional properties, bioactive compounds, biological properties

## Abstract

Research on bioactive compounds is essential to improve human health; promote adequate nutrition; drive innovation in the food, agricultural and biotechnology industries; and contribute to the preservation of the environment. The genus *Diplotaxis* (Brassicaceae) currently comprises around forty species, some of which are edible, particularly *Diplotaxis tenuifolia* (wild rocket), *Diplotaxis erucoides* (wall rocket), *Diplotaxis muralis* (annual wall rocket), *Diplotaxis viminea* (perennial wall rocket), and *Diplotaxis simplex*. The leaves of these species are rich in fiber and essential minerals, such as calcium, iron, potassium, and magnesium. Thirteen species have been characterized for their phenolic compounds, predominantly kaempferol, quercetin, and isorhamnetin glycosides. Furthermore, glucosinolate compounds were identified in nineteen species of the genus *Diplotaxis*. Many of the phytochemicals identified in *Diplotaxis* spp. demonstrated interesting biological activities, such as antioxidant, anti-inflammatory, antibacterial, hypoglycemic and hypolipidemic effects, as well as cytotoxicity and antiproliferative properties. This article provides a review of the phytochemistry of the *Diplotaxis* genus, highlighting its importance in food, its biological properties, potential pharmacological applications, and the dearth of research on many of these plants.

## 1. Introduction

In recent years, functional foods have emerged as a proactive approach to improve health through nutrition [1]. These foods contain essential nutrients as well as bioactive compounds that have beneficial effects on specific functions of the human body [2]. The use of aromatic, medicinal, and seasoning plants is an integral part of Iberian culture and a source of a variety of phytochemicals that are associated with various health benefits [3,4,5]. The incorporation of these plants into the diet not only adds flavor and aroma but also provides the body with a source of bioactive compounds that promote overall health and well-being [3,4]. Investigations of the phytochemistry and biological activity of these natural sources are essential to improve human health; promote adequate nutrition; drive innovation in the food, agricultural and biotechnology industries; and contribute to the preservation of the environment. Incorporating bioactive compounds into the diet may be a promising strategy for improving health and preventing disease [3,4,5]. However, it is also important to continue research to better understand the mechanisms of action and specific effects of these compounds on the human body, in addition to evaluating safety and effectiveness in different application contexts.

The Brassicaceae family is well-known for its abundance of bioactive compounds, including glucosinolates, which act as precursors to isothiocyanates, compounds linked to a range of health benefits. The genus *Diplotaxis* is part of this family. Some species of this genus are edible and widely used in cuisine, especially their leaves, which are consumed raw in salads or as ingredients in various dishes. Currently, the genus comprises approximately 40 species, with additional intraspecific taxa (Table 1).

This genus presents significant variation in geographic distribution, morphology, molecular markers, and chromosome number. It is found mainly in Europe in the Mediterranean region, particularly in its western part, which is considered the center of diversity; it is also found in the Iberian Peninsula and Southwest Asia, Northwest Africa and the Cape Verde archipelago [6,7]. At the taxonomic level, *Diplotaxis* species are mainly herbaceous annuals, with a group of perennial species (*D. ibicensis*, *D. tenuifolia*, *D. harra*, *D. antoniensis*, *D. glauca*, *D. gorgadensis*, *D. gracilis*, *D. hirta*, *D. sundingii*, *D. varia*, *D. vogelli*, *D. kohlaanensis* and *D. nepalensis*); the plants are classified as subshrubs with lignified branches at the base. The leaves are sessile and possess a pinnatifid or pinnatisect blade; however, in a few cases, they are subentire to shallowly lobed. The upper cauline leaves are sometimes different from the basal or median ones. The flowers are predominantly yellow within the genus but take on white hues in *D. erucoides* and violets in *D. acris* and *D. griffithii*, forming terminal racemes with fruiting racemes that are rather long. The seeds are typically quite small, ranging from 0.6 to 1.3 mm in length, and are ovate to ellipsoid in shape. However, it is worth noting that *D. siifolia* stands out for having spherical seeds [7]. 

**Figure 1:**
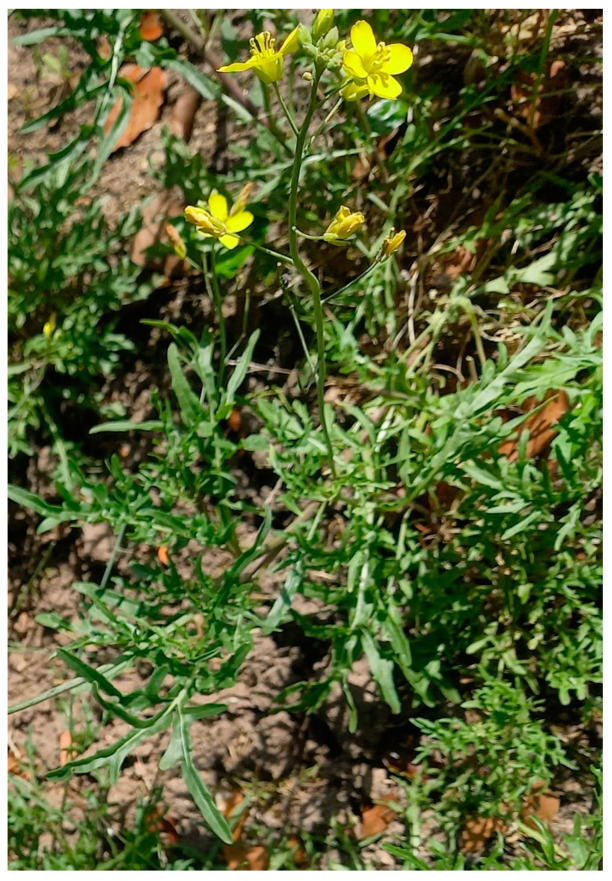
*Diplotaxis muralis* (L.) DC.

*Diplotaxis* species are generally diploid, with rare instances of polyploidy and notable dysploidy among species. The gametic numbers range from *n* = 7 in *D. erucoides* to *n* = 13 in the *D. harra* aggregate, with *D. muralis* serving as an exception (Figure 1). *D. muralis* (*n* = 21) is an allotetraploid derived from hybridization between *D. tenuifolia* (*n* = 11) and *D. viminea* (L.) DC (*n* = 10) [6,7,8,9]. 

Regarding agricultural production, *D. tenuifolia* stands out as the only species cultivated on a large scale within the genus, especially in Italy, where it occupies more than 4000 hectares. This country is a significant consumer of this plant, but its popularity is growing as a green salad in other parts of the world due to its spicy flavor [8,10]. While it is commonly grown in open fields, it is more frequently found in greenhouses. Additionally, *D. tenuifolia* can be cultivated without soil, using floating systems or the nutrient film technique. The leaves are harvested during the pre-flowering stage, between 20 to 100 days after planting or regrowth, depending on the cropping cycle, environmental conditions, and market demand. After the initial harvest, 4 to 5 additional cycles can occur, depending on soil and climate conditions. During hot periods, harvesting is preferably done in the morning to avoid leaf dehydration. Maintaining the cold chain, including the use of refrigerated trucks to transport products from the fields to processing facilities, is essential [8,10].

Regarding the application of the *Diplotaxis* genus in the food industry, several approaches can be considered. These plants can be used directly; the fresh leaves of edible plants are used in salads, pizza, sandwiches and soups or processed to extend shelf life and enhance versatility [11]. Additionally, the plant or/and extracts can be used to fortify foods or create nutraceuticals. These bioactive compounds offer health benefits beyond basic nutrition. A study has demonstrated the incorporation of dried plant material into bread for supplementation [12]. Regarding aromas in this genus, compounds formed by glucosinolates and their hydrolysis products play a crucial role in determining aroma and pungent flavors. Bitter or spicy flavors are attributed to glucosinolates, while a robust, acrid aroma results from the release of volatile isothiocyanates. Compounds such as dimeric glucosativin, progoitrin, and epiprogoitrin are recognized for their substantial role in shaping the distinctive flavor of rocket salad, a characteristic widely appreciated by consumers [13]. The compounds and aromas of these plants endow them with considerable potential. However, the choice of approach depends on the stability of the compounds during processing, consumer acceptance, the desired health benefits, as well as regulatory and market considerations. Despite its various uses and economic interest, an integrated review of the current knowledge about the *Diplotaxis* genus is lacking, especially in terms of its chemical composition and nutritional and biological properties. This review aims to bridge this gap by offering an updated and comprehensive overview that emphasizes their importance in nutrition, biological properties, and potential pharmacological applications as well as the limited research available on many of these plants. 

## 2. Methodology

For the present review, we collected papers published from 1990 to April 2024 and available in scientific electronic databases including Web of Science, Science Direct, PubChem, PubMed, Google Scholar, and ChemSpider. The scientific names and the distribution of *Diplotaxis* species were obtained from the WFO Plant list (www.wfoplantlist.org, accessed on 25 April 2024) and Plants of the World Online (www.plantsoftheworldonline.org, accessed on 25 April 2024) databases. 

## 3. Nutritional Properties

Some species of *Diplotaxis* are edible and frequently used in cooking, such as *D. tenuifolia* (wild rocket), *D. erucoides* (wall rocket), *D. muralis* (annual wall rocket), and *D. viminea* (perennial wall rocket); in particular, leaves from these species are consumed raw in salads or as ingredients in various dishes (e.g., pizza, sandwiches, and soups) [11]. 

*D. tenuifolia* leaves, *D. erucoides* leaves, and *D. simplex* leaves and flowers were analyzed for their macronutrient and minerals composition, with the results presented in Table 2 [8,14,15,16].

Plants from the *Diplotaxis* genus, such as *D. erucoides* leaves, *D. tenuifolia* leaves, and *D. simplex* leaves and flowers, are rich in carbohydrates, including fiber, and essential minerals, such as calcium, iron, potassium, and magnesium. Although the flowers exhibit higher levels of these constituents, the leaves are typically consumed due to the limited availability of flowers. Among the leaves of different species, *D. simplex* contains the highest quantities of these nutrients. However, *D. tenuifolia* is the only plant from the *Diplotaxis* genus currently widely cultivated and consumed. According to Regulation (EU) No 1169/2011 of the European Parliament and of the Council of 25 October 2011, the following daily doses of nutrients and minerals are recommended for adults: 260 g carbohydrates, 70 g lipids, 20 g acids saturated fats, 50 g protein, 800 mg calcium, 700 mg phosphorus, 375 mg magnesium, 14 mg iron, 10 mg zinc, and 1 mg copper [17].

No studies have been conducted on heavy metals to ensure the safety of these plants for consumption. The Brassicaceae family includes many metal-accumulating species. *Brassica juncea* can absorb and accumulate high levels of heavy metals, making it useful for phytoremediation of contaminated soils. Other species, such as *Brassica oleracea*, also accumulate heavy metals that can pose a health risk [18].

The concentrations of various lipid compounds, including fatty acids, in the leaves and flowers of *D. simplex* as well as in the flowers of *D. viragata* and the non-flowering aerial parts of *D. erucoides* were also analyzed and expressed as relative percentages, as shown in Table 3 [15,19].

*D. simplex* exhibited notably high levels of α-linolenic acid, with relative percentages of 27.7% in flowers and 25.4% in leaves. Phytol was also present at significant concentrations, with relative percentages of 17.6% in leaves and 3.7% in flowers. Additionally, other unsaturated fatty acids were identified in *D. simplex*, including oleic acid at 7.7% in leaves and 6.1% in flowers and linoleic acid at 5.6% in flowers and 4.4% in leaves, collectively comprising 39.4% of unsaturated fatty acids in flowers and 30.4% in leaves [15]. In *D. viragata*, unsaturated fatty acids comprised 42.8% of flowers, while in *D. erucoides*, they comprised 36.0% of the non-flowering aerial parts. Linoleic acid predominated, constituting 23.7% in *D. viragata* flowers and 29.1% in the non-flowering aerial parts of *D. erucoides* [19]. The consumption of unsaturated fatty acids benefits cardiovascular health and reduces inflammation [20].

*D. erucoides*, *D. tenuifolia*, and *D. simplex* exhibit nutritional value similar to that of *Eruca sativa* leaves, the commonly consumed arugula (rocket) [21,22]. Significant variations are observed in their mineral compositions and lipid profiles, specifically concerning fatty acids. It is important to note that data on the nutritional properties of the remaining species have not been reported, underscoring the necessity for further research in this area. Further studies are necessary to fully characterize the nutritional composition of *Diplotaxis* genus plants. Even for the most frequently utilized species, there remains a notable lack of comprehensive nutritional data. This emphasizes the critical need for additional research to gain a deeper understanding of the nutritional content and potential health advantages offered by the consumption of these plants.

## 4. Secondary Metabolites

**Figure 2:**
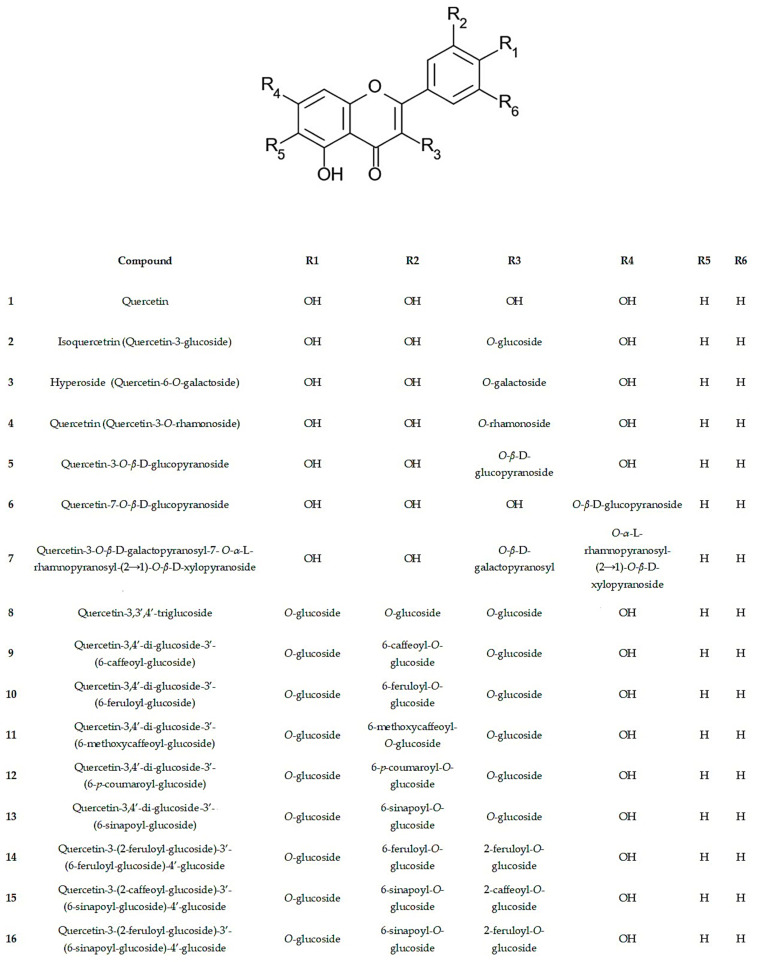

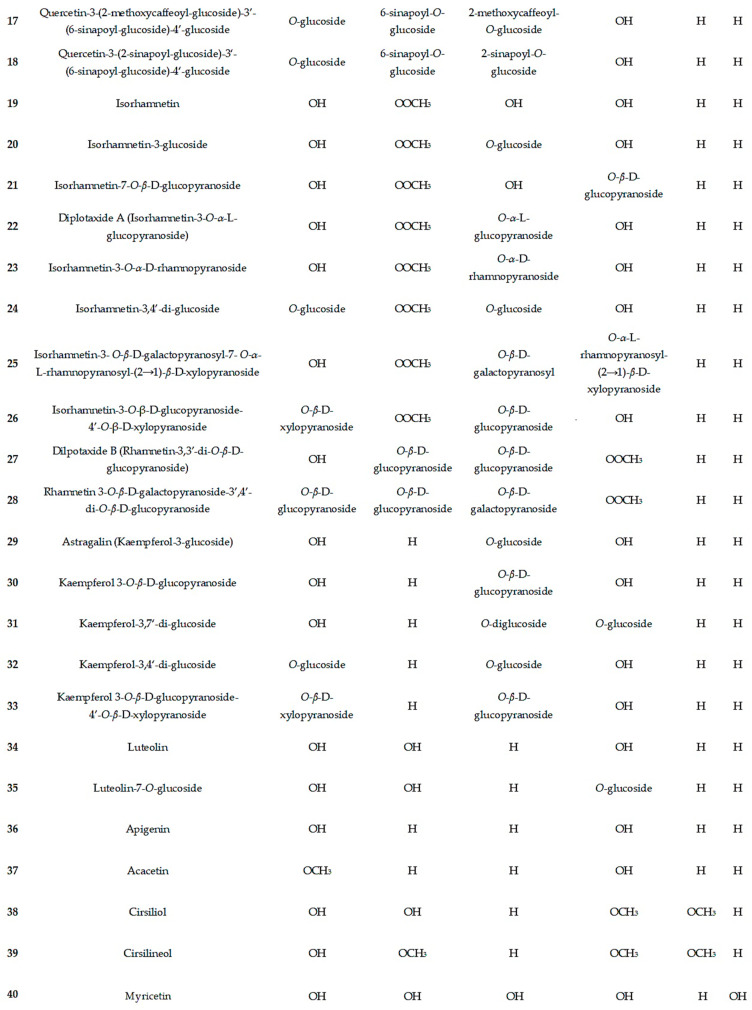

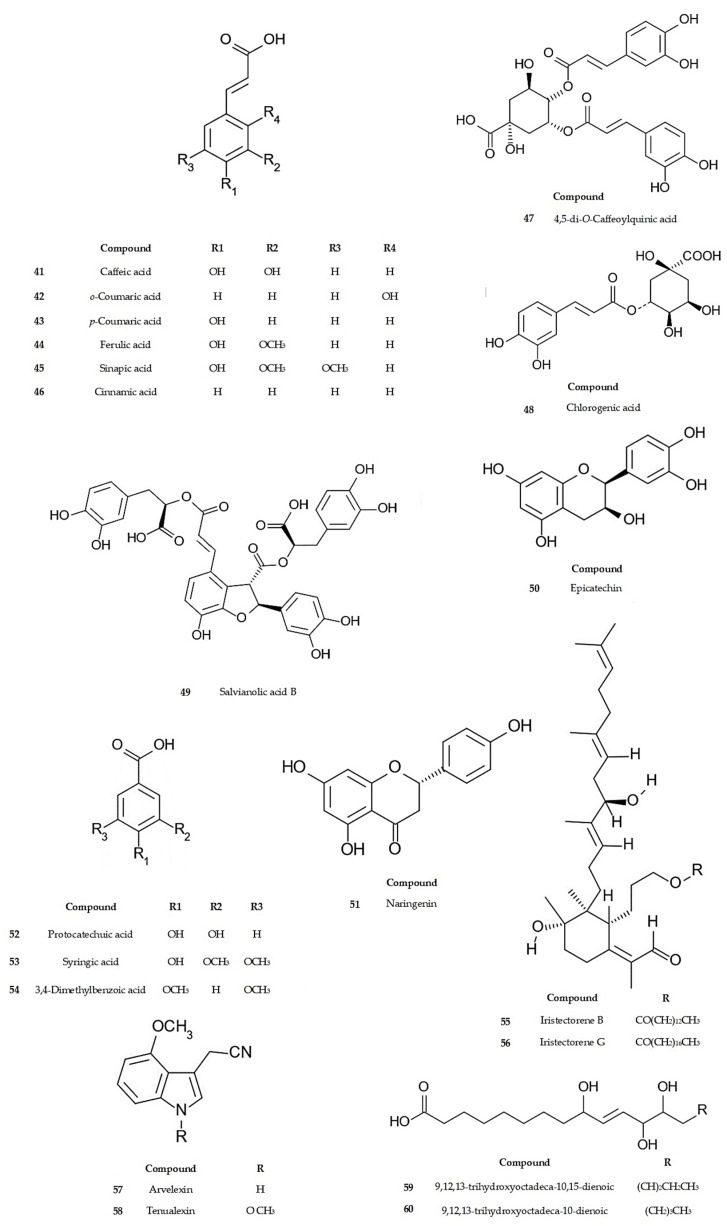

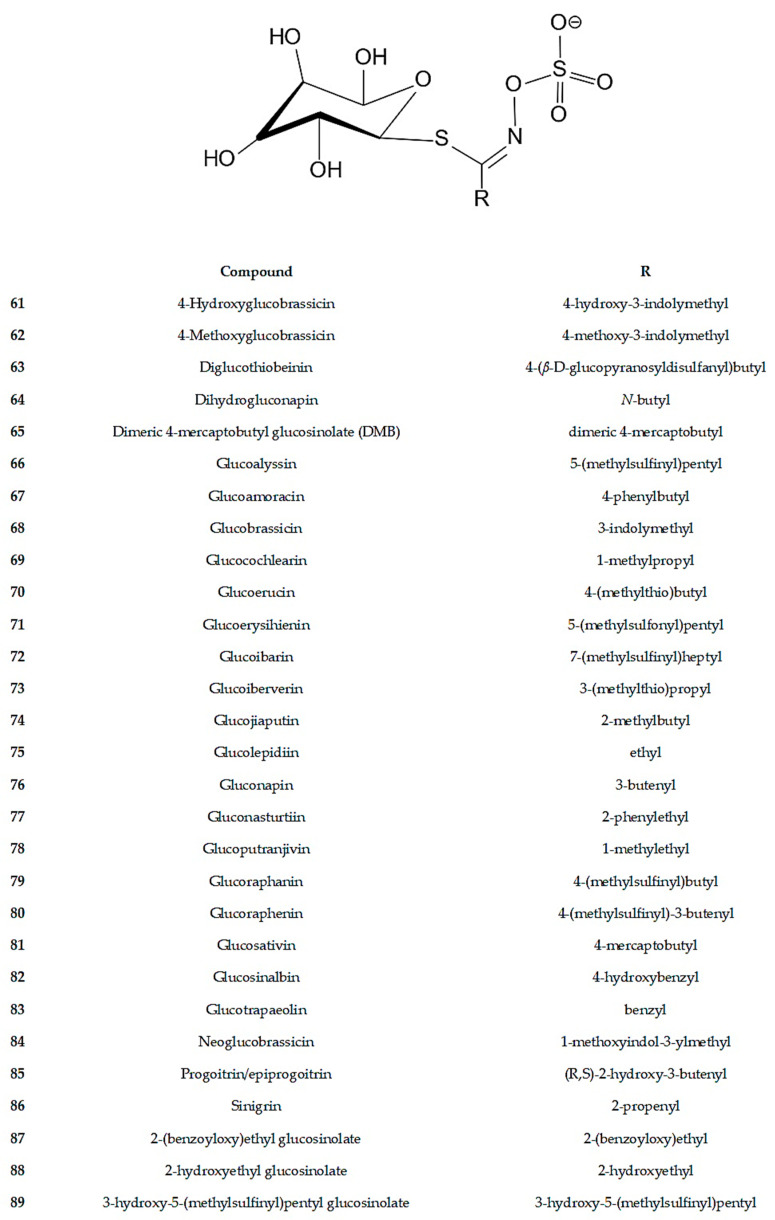
Compounds identified in the genus *Diplotaxis* (Compound **1** to **89**).

Among the 38 *Diplotaxis* species, only nineteen have undergone chemical characterization (Figure 2 and Table 4). Leaves and flowers emerged as the most frequently studied plant parts. The extraction process primarily utilized alcoholic solvents, predominantly methanol and ethanol at various concentrations. Only solid–liquid extractions were performed, with temperatures ranging from room temperature to 80 °C, without the use of advanced extraction technologies, such as pressurized liquids or supercritical fluids, or microwave/ultrasound-assisted extractions. Phenolic compounds and glucosinolates were the main classes of phytochemicals identified in the extracts. Glucosinolates were present in all parts of the plants studied, while phenolic compounds were identified in all parts except seeds and roots. Glucosinolates constitute a group of compounds commonly found in plants of the Brassicaceae family, particularly within the order Brassicales, and consequently within the genus *Diplotaxis*. To date, approximately 137 glucosinolates have been identified in plants, with 28 of them found within the genus *Diplotaxis*, among which glucolepidin, glucobrassicin, 4-hydroxyglucobrassicin, and 4-methoxyglucobrassicin are the most common [23,24,25]. To date, indol alkaloids and triterpenoids have been exclusively detected in the flowers of *D. simplex*, while oxylipins were found only in the aerial parts of *D. erucoides* [26,27]. Phenolic compounds identified in this genus primarily comprised flavonols and phenolic acids. Flavanones and flavanols, such naringenin in *D. simplex* and epicatechin in *D. simplex* and *D. harra,* were less frequently mentioned [12,28,29].

The first studies carried out on plants belonging to the *Diplotaxis* genus have unveiled the presence of flavonols. In *D. erucoides*, *D. erucoides* subsp. *longisiliqua*, *D. ibicensis*, *D. siettiana*, *D. assurgens*, *D. catholica*, *D. tenuisiliqua*, *D. virgata*, *D. vimínea*, *D. simplex*, *D. tenuifolia*, *D. harra*, and *D. muralis*, mono-, di-, and/or triglycosides of kaempferol, quercetin, and isorhamnetin (3-*O*; 7-*O*; and/or 3-*O*,7-*O*-glycosides) were identified, with glucose, galactose, and/or rhamnose representing the most frequent sugar moiety [9,30,31,32]. Although current technology did not exist at the time of these studies, generally the composition of flavonoids shows a great number of similarities between species and seems to indicate very close relationships. Additionally, the presence of phenolic acids, such as caffeic, *p*-coumaric, and ferulic acid derivatives, has been also documented [30]. With the advancement of instrumental analysis techniques, especially with the emergence of technologies such as HPLC-PDA, LC-MS and NMR, it is possible to achieve a more precise identification of compounds and chemical structures. The structural representations of the compounds identified using these methods are illustrated in Figure 2. Table 4 provides a compilation of *Diplotaxis* spp., their extracts, the plant parts used, and a summary of the identified compounds.

The species most extensively researched is *D. tenuifolia*, with numerous flavonol and glucosinolate compounds identified in methanolic leaf extracts [23,31,33,34,35]. Among these compounds, quercetin-3,3′,4-triglucoside (compound **8**) and quercetin-3,4′-diglucoside-3′-(6-sinapoylglucoside) (compound **13**) stand out as the most frequently reported flavonols [6,34]. While seeds and roots have also been characterized, there is a lack of studies on flowers [31].

In the ethanolic extract of *D. simplex* flowers, in addition to flavonols and glucosinolate compounds, triterpenoids (compounds **55** and **56**) and indolic alkaloids (compounds **57** and **58**) were also found [27,29]. However, in the methanolic extract, hydroxycinnamic and hydroxybenzoic acid compounds were identified [29]. Moreover, naringenin (compound **51**) was identified in the ethyl acetate:ethanol extract [28]. Additionally, flavonols, hydroxycinnamic acids, and hydroxybenzoic acids were identified in the ethanolic extract of leaves. It is noteworthy that flavanones and flavanols were also found [12]. Glucosinolates were exclusively reported in the leaves [25].

In the methanolic extract of *D. harra* flowers, flavonols and hydroxycinnamic acid compounds were identified. Glucosinolates were detected in leaves and seeds [23,29,31,33,34,35].

The alcoholic extract of *D. muralis* flowers isolated from an ethyl acetate fraction yielded the identification of quercetin-3-*O*-*β*-D-galactopyranosyl-7-*O*-*α*-L-rhamnopyranosyl-(2→1)-*O*-*β*-D-xylopyranoside (compound **7**) and isorhamnetin-3-*O*-*β*-D-galactopyranosyl-7-*O*-*α*-L-rhamnopyranosyl-(2→1)-*β*-D-xylopyranoside (compound **25**) [36]. Isorhamnetin-3-*O*-*α*-L-glucopyranoside (compound **22**) was identified in the *n*-butanol fraction obtained from ethanolic (80%) extracts of *D. virgata* flowers [19].

The ethanolic extract of the aerial parts of *D. erucoides* revealed the presence of several bioactive compounds, but these compounds have not been fully identified. These include derivatives of hydroxycinnamic acids, notably caffeic and coumaric acid derivatives, along with flavonol glycosides derived from quercetin, kaempferol, and isorhamnetin. However, glucosinolates were also identified, along with oxylipins **59** and **60** [26]. Compound **27** was identified in the *n*-butanol fraction obtained from an ethanolic (80%) extract of a non-flowering aerial part [19].

Only glucosinolates have been identified in *D. assurgens*, *D. berthautii*, *D. brachycarpa*, *D. brevisiliqua*, *D. catholica*, *D. cretacea*, *D. ibicensis*, *D. ilorcitana*, *D. ollivieri*, *D. siettiana*, *D. siifolia*, *D. tenusiliqua*, and *D. viminea*. Glucosinolates typically exist in an intact form within the vacuoles of various cell types. They undergo degradation into glucosinolate hydrolysis products through the action of an endogenous glycosylated thioglycosidase enzyme called myrosinase. Activation of myrosinase occurs following cell rupture, which can result from plant injuries, herbivore or insect feeding, or metabolism by intestinal bacteria, which release isothiocyanates, nitriles, thiocyanates, epithionitriles, indoles, oxazolidine-2-thiones, cyanopithioalkanes, ascorbigens, goitrogens, and epithioalkanes, upon hydrolysis [6,23]. Most of the biological activities of glucosinolates are linked to their hydrolyzed derivatives, and pharmacological studies have revealed biological activities including anti-inflammatory, antioxidant, and cholinesterase inhibitory effects, making them potential cancer preventive agents. In the food industry, these compounds find application in food preservation due to their ability to inhibit microbial growth [23].

**Table 4:** Compounds identified in different parts of the plant and extracts from the genus *Diplotaxis*.

Species	Part of Plant	Extracts/Fractions	Compounds	References
*D. assurgens*	Leaves	Methanol (70% + 10%)	Glucosinolates (**61**, **62**, **67**, **68**, **70**, **75**, **76**, **82**, **84**, **85**)	[25]
*D. berthautii*	Leaves	Methanol (70% + 10%)	Glucosinolates (**62**, **64**, **75**, **84**, **86**)	[25]
*D. brachycarpa*	Leaves	Methanol (70% + 10%)	Glucosinolates (**75**, **76**, **77**, **85**)	[25]
*D. brevisiliqua*	Leaves	Methanol (70% + 10%)	Glucosinolates (**86**)	[25]
*D. catholica*	Leaves	Methanol (70% + 10%)	Glucosinolates (**62**, **68**, **75**, **76**, **77**, **82**, **85**)	[25]
	Seeds	Methanol (100% + 70%)	Glucosinolates (**76**)	[37]
*D. cretacea*	Leaves	Methanol (70% + 10%)	Glucosinolates (**61**, **62**, **67**, **70**, **79**, **82**, **83**)	[25]
*D. erucoides*	Aerial part (per-flowering)	Ethanol (100%)	Oxylipins (**59**, **60**) Glucosinolates (**68**, **76**, **77**, **86**)	[26]
	Aerial part (non-flowering)	Ethanol (80%)/*n*-butanol	Flavonols (**27**)	[19]
	Flowers	Methanol (70%)	Glucosinolates (**70**, **79**, **81**)	[31]
	Leaves	Methanol (70%)	Glucosinolates (**62**, **70**, **79**, **81**)	[31]
		Methanol (70% + 10%)	Glucosinolates (**61**, **62**, **67**, **68**, **70**, **75**, **76**, **80**, **84**, **86**)	[25]
	Roots	Methanol (70%)	Glucosinolates (**62**, **70**, **79**, **82**)	[31]
	Seeds	Methanol (100% + 70%)	Glucosinolates (**73**, **82**)	[37]
		Methanol (70%)	Glucosinolates (**70**, **79**)	[31]
*D. harra*	Flowers	Methanol (100%)	Hydroxycinnamic acids (**41**, **45**, **48**), Flavanols (**50**)	[29]
	Leaves	Methanol (70% + 10%)	Glucosinolates (**61**, **62**, **66**, **67**, **68**, **75**, **76**, **79**, **82**, **84**, **86**, **88**)	[25]
	Seeds	Methanol (100% + 70%)	Glucosinolates (**82**)	[37]
*D. ibicensis*	Leaves	Methanol (70% + 10%)	Glucosinolates (**61**, **62**, **68**, **75**, **84**, **86**, **88**)	[25]
*D. ilorcitana*	Leaves	Methanol (70% + 10%)	Glucosinolates (**61**, **62**, **68**, **75**, **84**, **86**)	[25]
*D. muralis*	Flowers	Ethanol (80%)/ethyl acetate	Flavonols (**7**, **25**)	[36]
	Leaves	Methanol (70% + 10%)	Glucosinolates (**61**, **62**, **66**, **67**, **70**, **75**, **77**, **78**, **79**, **83**, **84**)	[25]
*D. ollivieri*	Leaves	Methanol (70% + 10%)	Glucosinolates (**61**, **62**, **68**, **70**, **75**, **82**, **84**, **85**, **86**)	[25]
*D. siettiana*	Leaves	Methanol (70% + 10%)	Glucosinolates (**61**, **62**, **68**, **75**, **84**, **86**)	[25]
*D. simplex*	Flowers	Methanol (100%)	Hydroxycinnamic acids (**41**, **43**, **48**) Flavanols (**50**) Hydroxybenzoic acids (**54**)	[29]
		Ethanol (100%)	Flavonols (**1**, **5**, **6**, **19**, **21**, **23**, **26**, **27**, **28**, **30**, **33**) Triterpenes (**55**, **56**) Alkaloids (**57**, **58**)	[27]
		Ethyl acetate:ethanol (1:1)	Flavonols (**1**, **3**, **4**, **34**, **36**, **37**, **38**, **39**) Hydroxycinnamic acids (**42**, **43**, **44**, **46**, **47**) Flavanone (**51**) Hydroxybenzoic acids (**52**, **53**)	[28]
	Leaves	Methanol (70% + 10%)	Glucosinolates (**61**, **62**, **64**, **66**, **67**, **68**, **70**, **77**, **79**, **83**, **84**)	[25]
	Leaves	Ethanol (100%)	Flavonols (**3**, **4**, **34**, **35**, **36**, **37**, **38**, **39**) Hydroxycinnamic acids (**43**, **44**, **49**) Flavanols (**50**) Flavanones (**51**) Hydroxybenzoic acids (**52**, **53**)	[12]
*D. siifolia*	Leaves	Methanol (70% + 10%)	Glucosinolates (**61**, **62**, **64**, **66**, **68**, **75**, **76**, **78**, **82**, **84**, **85**, **88**)	[25]
	Seeds	Methanol (100% + 70%)	Glucosinolates (**76**, **82**, **86**)	[37]
*D. tenuifolia*	Flowers	Methanol (70%)	Glucosinolates (**70**, **79**, **81**)	[31]
	Leaves	Methanol (50%)	Flavonols (**8**, **9**, **10**, **11**, **12**, **13**, **14**, **15**, **16**, **17**, **18**, **24**, **32**)	[23,34,35,38]
		Methanol (70%)	Flavonols (**2**, **8**, **9**, **13**, **20**, **29**, **24**, **31**, **32**, **40**) Glucosinolates (**61**, **62**, **63**, **65**, **66**, **70**, **72**, **75**, **73**, **79**, **80**, **81**, **83**)	[31,33]
		Methanol (70% + 10%)	Glucosinolates (**61**, **62**, **63**, **64**, **65**, **66**, **67**, **68**, **70**, **71**, **72**, **74**, **75**, **77**, **79**, **80**, **81**, **82**, **83**, **84**, **85**, **87**, **89**)	[23,25,35]
	Roots	Methanol (70%)	Glucosinolates (**62**, **70**, **79**, **82**)	[31]
	Seeds	Methanol (70%) Methanol (100% + 70%)	Glucosinolates (**70**, **79**)	[31,37]
*D. tenusiliqua*	Leaves	Methanol (70% + 10%)	Glucosinolates (**75**, **86**, **88**)	[25]
*D. viminea*	Leaves	Methanol (70% + 10%)	Glucosinolates (**67**, **75**, **84**, **88**)	[25]
	Seeds	Methanol (100% + 70%)	Glucosinolates (**69**, **78**, **86**)	[37]
*D. virgata*	Flowers	Ethanol (80%)/*n*-butanol	Flavonols (**22**)	[19]
	Leaves	Methanol (70% + 10%)	Glucosinolates (**61**, **62**, **67**, **68**, **70**, **75**, **76**, **77**, **82**, **84**, **85**, **86**, **88**)	[25]

## 5. Biological Properties

In this section, we will delve into the biological activities of the *Diplotaxis* genus, examining its antioxidant, anti-inflammatory, antibacterial, hypoglycemic and hypolipidemic activities, as well as cytotoxicity and antiproliferative properties. This exploration aims to unveil the broad spectrum of therapeutic potential within the *Diplotaxis* genus and its relevance in research.

### 5.1. Antioxidant Activity

The most extensively studied activity for various species of plants in the *Diplotaxis* genus is antioxidant activity. Many assays are currently available to evaluate different antioxidant effects such as radical scavenging using synthetic (DPPH or ABTS assays) or cellular radicals (superoxide, hydroxyl assays), reductive power (FRAP, molybdenum assays), and metal chelation. The antioxidant activity was assessed in extracts obtained from several plant parts of *D. simplex*, *D. harra*, *D. erucoides*, *D. virgata*, and *D. tenuifolia*. For all enumerated assays, negative controls were included.

*D. simplex* methanolic extracts and its fractions demonstrated antioxidant activity in different assays. Total antioxidant activity determined using the green phosphate/Mo^5+^ complex ranged from 17.3 to 30.44 mg gallic acid equivalents (GAE) per gram across different plant parts, with flowers displaying the highest activity. Using the iron reducing power method, EC_50_ values varied from 1187 to 1660 µg mL^−1^, with flowers also exhibiting better activity. Fractions obtained from methanolic extract of the flowers showed inhibition percentages between 4.82% and 27.43% in the ABTS assay and 25.17% to 75.99% in the β-carotene method, with the 60% methanolic fraction exhibiting the best inhibition percentages in both assays. The antioxidant activity was observed, at least in part, in the phenolic compounds found in the extracts, which displayed a total phenolic content (TPC) ranging from 2.86 to 7.65 mg GAE g^−1^ across plant parts and a total flavonoids content (FC) ranging from 2.11 to 3.93 mg (+)-catechin equivalent (EC) per gram, with flowers showing the highest content in both assays. Regarding total condensed tannins (CTCs), values ranged from 0.61 to 0.81 mg EC g^−1^, with leaves having the highest value [29]. In another study, the ethanol extract obtained from flowers exhibited compelling antioxidant potential across various assays. It exhibited a noteworthy EC_50_ of 0.10 mg mL^−1^ in the reducing power assay, an IC_50_ of 0.20 mg mL^−1^ in the DPPH scavenging activity assay, and an IC_50_ of 12.50 mg mL^−1^ in the *β*-carotene bleaching assay. Regarding the Fe^2+^ chelating capacity, the ethyl acetate extract demonstrated better antioxidant activity with an IC_50_ of 0.20 mg mL^−1^, and the ethanol extract had a higher value of 0.60 mg mL^−1^. The observed antioxidant activity could be attributed largely to phenolic content, as evidenced by a TPC of 17.10 mg GAE g^−1^ for the ethyl acetate extract and 52.70 mg GAE g^−1^ for the ethanolic extract. Regarding flavonoids, 100.60 mg of quercetin equivalents (QE) per gram was measured in the ethyl acetate extract, while 74.20 QE g^−1^ was noted in the ethanol extract [39]. In another study using aqueous extracts from roots, stems, leaves, flowers, and siliques, the ABTS and DPPH assay showed IC_50_ values ranging from 0.35 to 1.68 mg mL^−1^ and 0.31 to 15.91 mg mL^−1^, respectively, with seeds demonstrating better activity in both methodologies. For the superoxide assay, IC_50_ values ranged from 0.46 to 2.60 mg mL^−1^, with seeds having the lowest value. The hydroxyl assay displayed IC_50_ values between 0.49 and 1.17 mg mL^−1^, with flowers exhibiting the lowest value. For the NO assay, IC_50_ values ranged from 1.37 to 38.47 mg mL^−1^, with seeds exhibiting better activity. In the chelating activity assay, IC_50_ values varied from 0.41 to 6.29 mg mL^−1^, with leaves showing the lowest value. Although antioxidant activity was observed, the phenolic profile of the seeds was not characterized. Finally, the molybdate assay displayed values between 12.34 and 38.76 mg ascorbic acid equivalent (AAE) 100 g^−1^, with the siliques showing the highest value. The presence of phenolic compounds within the extract may largely account for its antioxidant activity, as evidenced by a TPC ranging from 446.8 mg to 3433.4 mg caffeic acid equivalent (CAE) per gram, a FC ranging from 344.8 mg to 2422.4 mg CAE 100 g^−1^, and di-hydroflavonol content ranging from 24.1 mg to 119.8 mg CAE 100 g^−1^, dry weight [40].

In aqueous extracts of the flowering aerial parts of *D. harra*, an IC_50_ value of 2.11 mg mL^−1^ was obtained with the DPPH assay [41]. The ethanolic extract (95%) from aerial parts (pre-flowering) demonstrated significant antioxidative activity, with IC_50_ values of 247.4 µg mL^−1^ for the ABTS assay and 203.7 µg mL^−1^ for the DPPH assay, along with a phenol content of 80.43 mg GAE g^−1^ and a flavonoid content of 54.26 mg QE g^−1^ [42]. In another study, antioxidant evaluation was also conducted using separated leaves, stems, flowers, siliques, seeds, and roots. In the ABTS assay, different parts of the plant showed IC_50_ values ranging from 0.35 to 4.13 mg mL^−1^, with seeds displaying an IC_50_ of 0.35 mg mL^−1^ against ABTS radicals. The high antioxidant activity exhibited by the seeds is noteworthy, especially considering that glucosinolates were the only compounds identified. Although glucosinolates have some antioxidant activity, it is likely that other compounds with antioxidant activity not identified in this study may be present. Consequently, additional research on chemical composition is needed. For the DPPH assay, IC_50_ values varied from 0.86 to 13.06 mg mL^−1^. For the superoxide assay, IC_50_ values ranged from 0.79 to 4.13 mg mL^−1^). Here, the flowers demonstrated the highest antioxidant activity. The seed extract exhibited more activity in the hydroxyl (IC_50_ values ranged from 0.48 to 1.30 mg mL^−1^) and chelating (IC_50_ values ranged from 1.58 to 3.81 mg mL^−1^) assays. Regarding nitric oxide (NO) (IC_50_ values varied from 1.18 to 5.92 mg mL^−1^) and molybdate (22.50 to 29.46 mg AAE 100 g^−1^ dry weight), the extract from the leaves was the most active. This antioxidant activity may be largely due to the presence of phenolic compounds in the extract as it exhibited a TPC that varied from 383.7 to 2694.5 mg CAE 100 g^−1^ dry weight across plant parts. The seeds exhibited the highest amount, while leaves showed the lowest. Although the presence of reducing compounds was observed, the phenolic profile of the seeds was not characterized. The flavonol content ranged from 383.7 to 1922.6 mg CAE 100 g^−1^ across plant parts, with seeds having the highest and leaves the lowest. Di-hydroflavonols content varied from 70.8 to 149.8 mg CAE 100 g^−1^, with flowers showing the highest content [40]. In another study using methanolic extracts from flowers, leaves, and stems, the antioxidant activity, which was determined using phosphate/Mo^5+^ complex assays, ranged from 16.14 to 62.11 mg GAE g^−1^ in dry weight, with the flower extract exhibiting the highest activity. The fractions obtained from flower extract exhibited between 1.48% and 35.05% ABTS inhibition and between 13.66% and 77.15% *β*-carotene assay inhibition. The 60% aqueous methanol fraction demonstrated the highest inhibitory percentages in both assays. The presence of phenolic compounds in the extract could explain much of its antioxidant activity, with a TPC ranging from 1.27 to 11.11 mg GAE g^−1^ across plant parts and flowers exhibiting the highest TPC. The total flavonoid content ranged from 1.12 to 5.69 mg EC g^−1^, with flowers showing the highest value. Regarding CTC, values ranged from 0.68 to 1.75 mg EC g^−1^, with leaves having the highest value [29].

The ethanolic extract from the pre-flowering aerial parts of *D. erucoides* revealed antioxidant activity in several assays, such as ABTS (IC_50_ value of 97.87 μg mL^−1^), DPPH, and FRAP assays, with IC_50_ values of 135.13 μg mL^−1^ and 16.88 μMFe(II) g^−1^, respectively. In the *β*-carotene assay, the extract exhibited interesting IC_50_ values of 10.32 μg mL^−1^, indicating effective protection against lipid peroxidation after 60 min. Positive controls were included in this assay. Ascorbic acid yielded IC_50_ values of 5.0 ± 0.82 μg mL^−1^ and 1.7 ± 0.06 μg mL^−1^ in the DPPH and ABTS assays, respectively. BHT achieved an IC_50_ value of 63.2 ± 2.30 μMFe (II) g^−1^ in the FRAP assay, while propyl gallate demonstrated an IC_50_ of 0.09 ± 0.04 μg mL^−1^ in the β-carotene assay. Compared to the positive controls, the extracts exhibit some antioxidant activity. This antioxidant activity may be largely due to the presence of phenolic compounds. In fact, the extract exhibited a TPC of 348.8 mg of chlorogenic acid equivalents per 100 g of plant material and a FC of 111.1 mg QE 100 g^−1^ of plant material [26]. Another study used ethanolic extracts of aerial parts but proceeded to fractionate them with ethyl acetate and *n*-butanol to evaluate antioxidant activity in DPPH and ABTS assays [19]. The ethanolic (80%) extract from the non-flowering aerial parts of *D. erucoides* showed an IC_50_ of 27.02 µg mL^−1^ for the DPPH assay and 27.04 µg mL^−1^ for the ABTS assay. The ethyl acetate fraction obtained an IC_50_ of 26.03 µg mL^−1^ for the DPPH assay and 32.06 µg mL^−1^ for the ABTS assay. The *n*-butanol fraction displayed an IC_50_ of 24.01 µg mL^−1^ for the DPPH assay and 28.05 µg mL^−1^ for the ABTS assay. In this case, much of the antioxidant activity can be attributed to the presence of phenolic compounds since compound **27**, isolated from the *n*-butanol fraction, exhibited IC_50_ values of 18.00 µg mL^−1^ for the DPPH assay and 19.04 µg mL^−1^ for the ABTS assay, showing better antioxidant activity in relation to DPPH than its aglycone, ramnetin, which has an IC_50_ value of 140 μg mL^−1^ [19]. The fractions exhibit activity similar to the extract, as does the isolated compound. This certainly suggests that some of the activity can be attributed to these compounds.

*D. virgata* flowers extracts also contain compounds with antioxidant activity. An ethanol extract (80%) exhibited IC_50_ values of 26.03 µg mL^−1^ and 31.80 µg mL^−1^ for the DPPH and ABTS assays, respectively. Its fractions also had antioxidant activity. The *n*-butanol fraction, which displayed the highest activity, exhibited IC_50_ values of 20.01 µg mL^−1^ and 25.54 µg mL^−1^ for the DPPH and ABTS assays, respectively [19]. It is noteworthy that isorhamnetin glycoside (compound **22**), isolated from the *n*-butanol fraction, demonstrated even more significant antioxidant activity, with IC_50_ values of 16.01 µg mL^−1^ for the DPPH and 17.03 µg mL^−1^ for the ABTS assays. Compound 22 is more active in the DPPH assay than its aglycone, isorhamnetin, which has an IC_50_ value of 24.5 μg mL^−1^ [19]. There have been no studies conducted on antioxidant activity using other parts of the plant, indicating the necessity for broader investigations. This species requires further evaluation of its activity on cellular radicals (superoxide, hydroxyl), as well as its reducing and chelating capabilities.

*D. tenuifolia* exhibited FRAP values ranging from 4.13 mmol kg^−1^ to 11.02 mmol kg^−1^ fresh weight (fw) in aqueous leaf extracts. This significant antioxidant activity could potentially be attributed to the phenolic compounds present, namely flavonoids, with quercetin levels ranging from 0.68 to 7.74 mg 100 g^−1^ and lutein levels ranging from 40.92 to 54.48 μg g^−1^ fw upon hydrolysis [43]. In another study, aqueous extracts yielded a 29.30 mg CAE g^−1^ [44]. The evaluation of the reducing activity of this species has been conducted; however, assays are still needed to demonstrate its ability to scavenge radicals, known as scavenging activity.

In the Brassicaceae family, including white cabbage, broccoli, Italian kale, savoy cabbage, green cauliflower, cauliflower, and Brussels sprouts, the EC_50_ values range from 81.45 to 917.81 mg of sample per mg of DPPH, in dry weight. The total phenolic content ranges from 4.30 to 13.80 mg GAE g^−1^, the flavonoid content ranges from 1.35 to 6.71 mg CE g^−1^, and the tannin content ranges from 0.38 to 0.56 mg CE g^−1^ [45]. These values align with those obtained in various studies of *Diplotaxis* spp., and slightly higher antioxidant activity is occasionally observed in the DPPH assay. In *D. harra*, the activity is significantly enhanced. The ethanolic extract (95%) of aerial parts harvested during the pre-flowering stage shows an exceptional accumulation of bioactive compounds, indicating heightened activity during this growth phase. Nonetheless, the antioxidant standards demonstrate significantly superior activity, with EC_50_ values of 1.18 μmoles mg^−1^ DPPH for ascorbic acid, 2.80 μmoles mg^−1^ DPPH for kaempferol, 1.15 μmoles mg^−1^ DPPH for quercetin, and 2.03 μmoles mg^−1^ DPPH for quercitrin [45]. The total phenolic and flavonoid contents in different studies of *Diplotaxis* spp. generally fall within the range observed in other Brassicaceae species, occasionally exhibiting slightly lower values [45].

However, phenolic compounds are not exclusively responsible for antioxidant activity. Glucosinolates and respective hydrolysis products also have antioxidant activity. This is the case for sinigrin and its allyl isothiocyanates studied in *Brassica juncea* L., *Brassica rapa* L., and curly kale leaves. Additionally, gluconapine was studied in *Brassica juncea* L. and *Brassica rapa* L.; glucoalyssin and progoitrin were studied in *Brassica rapa* L.; and glucobrassicin was studied in *Brassica rapa* L., *Isatis canescens*, and curly kale leaves [46].

Beyond the few species studied, there are gaps in antioxidant activity research. Only some species have been subjected to assays with cellular radicals; in the other species, the assessment has been limited to synthetic radicals (DPPH and ABTS). Additionally, in many cases, assays are lacking to evaluate other antioxidant activity mechanisms, such as chelation and reduction.

### 5.2. Anti-Inflammatory Activity

Antioxidant activity is directly related to the inflammatory mechanism, with inflammation often triggered by oxidative stress and characterized by an imbalance between the production of reactive oxygen species (ROS) and the body’s antioxidant system’s capacity to neutralize them [47].

Notably, there are only two studies investigating the anti-inflammatory activity within the *Diplotaxis* genus. Ethyl acetate and ethanol extracts from *D. simplex* flowers demonstrated inhibition of phospholipase A2 activity, with an IC_50_ value of 2.97 mg ml^−1^, while the ethanolic extract exhibited an IC_50_ of 5 mg ml^−1^. In this assay, a negative control was included. Albino mice were administered the extracts, at 200 mg kg^−1^, via intraperitoneal injection before the induction of edema by carrageenan. This resulted in a reduction of paw edema in mice, with the diameter decreasing from 0.38 cm to 0.24 cm for the ethyl acetate extract and from 0.38 cm to 0.25 cm for the ethanol extract, and these effects were observed 4 h after the carrageenan challenge. In comparison, the negative control group, which received a normal saline solution containing 0.90% (m/v) NaCl, showed a change in diameter from 0.36 cm to 0.37 cm, while the positive control group, administered an intraperitoneal injection of indomethacin (50 mg/kg), exhibited a diameter change from 0.24 cm to 0.25 cm after 4 h [39]. However, the intraperitoneal administration route used in the in vivo assay, rather than oral, does not allow for the validation of the activity under normal conditions of plant use.

In *D. harra*, the ethanolic extract (95%) of arial parts (per-flowering) demonstrates the ability to inhibit the expression of inducible nitric oxide synthesis (iNOS) and cyclooxygenase-2 (COX-2) in RAW 264.7 macrophages stimulated with lipopolysaccharides (LPSs) by approximately 28.51% and 30.19%, respectively, at a concentration of 20 µg mL^−1^. Dexamethasone (DEX) was used as a positive control. The isolated compound, isorramnetin-3-*O*-*β*-D-glucoside, exhibited potent anti-inflammatory activity, notably suppressing the overexpression of iNOS by about 35.96% and 29.34%, respectively, and these effects are similar to those noted for DEX at 10 µM [42].

Although preliminary data on the anti-inflammatory activity of the genus *Diplotaxis* are promising, more comprehensive studies, including in vivo trials and detailed analyses of its mechanisms of action, are essential for a complete understanding of the therapeutic potential of these extract. Other aspects to highlight include the limited number of species studied, few experimental models, and unidentified bioactive compounds.

### 5.3. Antibacterial Activity

Antioxidant activity provides an intriguing starting point for investigating how natural compounds not only combat oxidative damage but also possess antimicrobial properties, highlighting the close relationship between these two activities [48]. Antibacterial activity was reported only for *D. virgata*, *D. erucoides*, *D. harra,* and *D. simplex*.

In *D. virgata*, the antibacterial activities of *n*-butanol fractions of flowers obtained from ethanolic (80%) extracts and isolated flavonol (**22**) were investigated in vitro against Gram-positive bacteria (*Listeria monocytogenes* ATCC 11120 and *Staphylococcus aureus* ATCC 25923) and Gram-negative bacteria (*Aeromona hydrophila* ATCC 1943, *Pseudomonas aeruginosa* ATCC 9027, *Salmonella enteritidis* ATCC 14028, *Escherichia coli* ATCC 25922, and *Klebsiella pneumonia* ATCC 13833) using the disk diffusion method. The positive control used discs impregnated with an ampicillin solution (5 mg/mL), while a negative control was also performed. The *n*-butanol fraction exhibited inhibition diameters of 20.00 mm and 19.04 mm in Gram-positive bacteria *L. monocytogenes* and *S. aureus*, respectively. In Gram-negative bacteria, its inhibition diameters ranged from 15.00 to 22.03 mm, with the highest value observed for *E. coli*. Similarly, the flavonol (compound **22**) exhibited inhibition diameters of 13.60 mm and 12.04 mm against *L. monocytogenes* and *S. aureus*, respectively. In Gram-negative bacteria, its inhibition diameters ranged from 12.50 mm to 16.55 mm, with the highest value observed also for *E. coli*. Comparatively, the inhibition diameters for ampicillin were as follows: 16.00 mm for *L. monocytogenes*, 18.00 mm for *S. aureus*, 17.00 mm for *A. hydrophila*, 19.00 mm for *P. aeruginosa*, 19.00 mm for *S. enteritidis*, 20.00 mm for *E. coli*, and 21.00 mm for *K. pneumoniae* [19].

The antibacterial activities of the *n*-butanol fractions derived from the non-flowering aerial parts of *D. erucoides* and the isolated compound **27** were assessed against the same bacterial strains and controls. The *n*-butanol fraction exhibited inhibition diameters of 16.02 mm and 20.04 mm in Gram-positive bacteria *L. monocytogenes* and *S. aureus*, respectively. In Gram-negative bacteria, its inhibition diameters ranged from 16.00 to 21.07 mm, with the highest value observed for *A. hydrophila*. The flavonol (compound **27**) exhibited inhibition diameters of 14.50 mm and 13.00 mm against *L. monocytogenes* and *S. aureus*, respectively. In Gram-negative bacteria, its inhibition diameters ranged from 14.00 mm to 17.60 mm, with the highest value observed for *E. coli* [19].

In *D. harra*, fractions of deslipidified methanolic flower extract were assessed to determine their antibacterial efficacy against *Pseudomonas aeruginosa* ATCC 27853, *Escherichia coli* ATCC 35218, and *Micrococcus luteus* NCIMB 8166. The 60% aqueous methanol fraction exhibited notable efficacy against *P. aeruginosa*, showcasing a 34% inhibition rate, while other fractions displayed comparatively lower inhibition percentages, ranging from 0% to 8%. In the case of *E. coli*, the 60% and 80% aqueous methanol fractions demonstrated the highest inhibition percentages at 48% and 34% inhibition, respectively, while the remaining fractions exhibited inhibition rates ranging from 0% to 11%. Regarding *M. luteus*, the 60% aqueous methanol fraction displayed the highest inhibition percentage at 53%, with the remaining fractions showing inhibition rates ranging from 8% to 26% [29]. In this assay, a microbial suspension and an antibiotic mixture containing 5 mg mL^−1^ streptomycin and 10 mg mL^−1^ penicillin G were used as controls. For *D. harra*, there is no information available regarding the compounds responsible for the activity.

The fractions of deslipidified methanolic flower extract of *D. simplex* were similarly evaluated across different concentrations against the same bacterial strains. In the case of *P. aeruginosa*, the 60% aqueous methanol fraction stood out with a significant inhibition rate of 17%, while other fractions displayed inhibition rates ranging from 0% to 14%. Concerning *E. coli*, the 80% fraction exhibited substantial inhibition at 93%, with other fractions ranging from 8% to 26%. Notably, the 80% aqueous methanol fraction displayed remarkable inhibition against *M. luteus*, reaching 100%. The 60% aqueous methanol fraction showed an inhibition value of 40%, and the remaining fractions demonstrated inhibition rates varying from 14% to 24%. In this assay, a microbial suspension and an antibiotic mixture containing 5 mg mL^−1^ streptomycin and 10 mg mL^−1^ penicillin G were used as controls [29]. For *D. simplex*, there is no information available regarding the compounds responsible for the activity.

### 5.4. Hypoglycemic and Hypolipidemic Activity

Metabolic diseases represent a diverse group of conditions that disrupt the biochemical and energy balance of the body [49]. The quest for compounds with hypoglycemic and hypolipidemic properties has been an area of interest in biomedical research. However, to date, only three species of the *Diplotaxis* genus have been subjected to studies on these activities.

An ethanolic extract of *D. erucoides* was evaluated for its ability to inhibit carbohydrate hydrolyzing enzymes. It showed slightly higher activity against *α*-glucosidase (IC_50_ = 85.18 μg mL^−1^) than *α*-amylase (IC_50_ = 92.36 ± 4.31 μg mL^−1^). In both tests, acarbose was used as a positive control, yielding IC_50_ values of 35.5 ± 1.22 μg mL^−1^ and 50.0 ± 0.93 μg mL^−1^, respectively [26]. Inhibiting *α*-amylase, which is crucial for starch and glycogen digestion, is vital in managing conditions like diabetes. Similarly, targeting *α*-glucosidase in the brush border membrane of the small intestine is essential. By hindering its function, the breakdown of disaccharides into glucose slows, effectively regulating sugar influx into the bloodstream and maintaining insulin levels. This inhibition consequently reduces carbohydrate digestion rates, contributing to lower bloodstream sugar levels and suppressing postprandial hyperglycemia [50,51,52]. Moreover, the same extract exhibited inhibitory effects on pancreatic lipase with an IC_50_ value of 61.27 μg mL^−1^, in comparison to the positive control orlistat, which exhibited an IC_50_ value of 37.1 ± 1.30 μg mL^−1^ [26]. In addition, the ethanolic extract of *D. tenuifolia* obtained an IC_50_ value of 7.76 ± 0.08 mg mL^−1^, in comparison to the positive control orlistat, which obtained an IC_50_ value of 0.018 ± 0.001 mg mL^−1^ [44]. The inhibition of pancreatic lipase may hold potential interest in the treatment of dyslipidemias. Pancreatic lipase is an enzyme secreted by the pancreas that breaks down triglycerides, the main lipids in the diet, into fatty acids and glycerol, which are then absorbed by the small intestine. When pancreatic lipase activity is inhibited, fat digestion and absorption are reduced [53,54].

The ethyl acetate extract of *D. simplex* flowers was also tested using inhibition assays of *α*-amylase and *α*-glucosidase. In both tests, acarbose was used as a positive control. The extract was more effective in inhibiting *α*-glucosidase (IC_50_ = 0.046 mg ml^−1^) than *α*-amylase (IC_50_ = 3.46 mg ml^−1^) [15]. Inhibiting *α*-glucosidase may be more advantageous in patients with diabetes, as this enzyme is directly involved in the final breakdown of complex carbohydrates into glucose, thereby directly affecting postprandial blood sugar levels [50,51,52]. Ethyl acetate and ethanol extracts from *D. simplex* flowers were assessed for their ability to inhibit rHPL (recombinant human pancreatic lipase) activity. A control sample was prepared without plant extract. The ethyl acetate extract demonstrated a more potent inhibition, with an IC_50_ value of 0.14 mg/mL, compared to the ethanol extract, which showed an IC_50_ of 0.32 mg ml^−1^ for pancreatic lipase activity [28]. The in vivo inhibitory effect on postprandial hyperglycemia was investigated using an ethyl acetate extract of *D. simplex* flowers administrated by gastric gavage. This extract was able to inhibit the rise in blood glucose levels in maltose-loaded mice, in a similar manner to treatment with the standard antihyperglycemic agent acarbose [15]. The protective effect of the ethyl acetate extract of *D. simplex* was confirmed in vivo, where administering 200 mg kg^−1^ of this extract for 30 days restored normal glycemia and serum lipid profiles in alloxan-induced diabetic rats and confirmed the protection of the pancreas, liver, and kidneys against damage caused by hyperglycemia [27].

Beyond the paucity of in vivo studies, the authors have conducted only a limited number of phytochemical studies to identify the compounds responsible for the observed activities. This gap may restrict our understanding of the underlying mechanisms behind the activity of the evaluated plants. Conducting further phytochemical studies to identify and characterize the active components present in these plant species would be highly beneficial.

### 5.5. Cytotoxicity and Antiproliferative Activity

Cytotoxicity and antiproliferative activity were studied to determine their ability to cause cellular damage or induce cell death, as well as to analyze their capacity to inhibit cell proliferation, thereby slowing down or preventing cell growth and multiplication. This study is relevant in the context of developing treatments for diseases such as cancer. Only two species from the *Diplotaxis* genus have been subject to studies concerning these activities.

The cytotoxicity and antiproliferation activity of the leaf aqueous extract of *D. tenuifolia* were study using human colon carcinoma cells (Caco-2 cells). The cytotoxic effect was evaluated using the 3-(4,5-dimethylthiazol-2-yl)-2,5-diphenyltetrazolium bromide (MTT) test to measure cell viability. To evaluate the anti-proliferative activity in cells, an analysis of cell cycle distribution was performed, determining the incorporation of bromodeoxyuridine (BrdU) in relation to DNA content. In Caco-2 cells, only concentrations of 50 and 100 mL L^−1^ reduced cell viability to values between 71% and 29%, respectively. Furthermore, the extract influenced cell proliferation; Caco-2 cells treated with 10 mL/L showed a significant accumulation in the G1 phase, compared to the control cells, and a corresponding reduction in the S and G2 + M phases, as determined using DNA content cytometric analysis [43]. These results indicate a decrease in DNA synthesis and a slowdown in cell cycle progression. This suggests that the extract may be inhibiting the cell’s ability to divide and proliferate, a crucial aspect in cancer treatment research, where suppressing cell proliferation is an essential therapeutic goal.

Ethyl acetate and ethanol extracts from *D. simplex* flowers were also studied against the human colon cancer cell line Caco-2 using the MTT assay. A control without plant extract was included in this assay. These extracts were effective in inhibiting cancer cell growth, with IC_50_ values of 62.00 and 63.25 μg mL^−1^ for ethanolic and ethyl acetate, respectively [39]. Identical extracts and methodology were employed to examine their impact on MCF7 human breast cancer cells and K562 human leukemia cells. The flower extracts exhibited IC_50_ values ranging between 90 and 150 µg ml^−1^ for the inhibition of MCF7 cells. Notably, the ethyl acetate extract showed significant efficacy in inhibiting K562 human leukemia cells with an IC_50_ value of 20 µg ml^−1^, compared to the ethanolic extract with an IC_50_ of 100 µg ml^−1^ [28].

The same comment applies to these studies. Only a limited number of species were investigated, with the authors conducting only a few phytochemical analyses to identify the compounds responsible for the observed activities. This limitation could hinder our understanding of the underlying mechanisms of the activity observed in the evaluated plants. Conducting additional phytochemical studies to identify and characterize the active components present in these plant species would be highly beneficial.

## 6. Conclusions

Within the *Diplotaxis* genus, only a minority of its 38 species have undergone thorough phytochemical analysis and assessment of their biological properties. Identified compounds predominantly include flavanols, mainly mono-, di-, and/or triglycosides of kaempferol, quercetin, and isorhamnetin (3-*O*; 7-*O*; and/or 3-*O*,7-*O*-glycosides), with glucose, galactose, and/or rhamnose as the predominant sugar moieties. Additionally, glucosinolates, triterpenoids, indolic alkaloids, and oxylipins have been identified. Despite this limited focus, the *Diplotaxis* genus still stands out as a promising source of compounds with notable nutritional and biological properties. Its functional attributes range from biological activities such as antioxidant, anti-inflammatory, antibacterial, hypoglycemic, and hypolipidemic effects to cytotoxic and antiproliferative properties. Although nearly all trials have been conducted in vitro, it is crucial to conduct further in vivo studies and clinical trials to validate the results and determine the therapeutic potential of these plants. However, there remains a gap in identifying the phytochemical composition of the extracts and understanding their significance. It is noteworthy that among the activities studied, antioxidant activity has received the most attention. Furthermore, the contribution of glucosinolates and their respective isothiocyanates in the extracts is often overlooked, despite some of their biological activities being known, as described in the genus. Future research endeavors are necessary to deepen our understanding of the *Diplotaxis* genus, elucidating the mechanisms behind these properties. Quantitative analyses of secondary metabolites are particularly needed. Such investigations will not only expand our knowledge but also pave the way for the development of novel applications and products derived from *Diplotaxis* species, potentially leading to the creation of innovative functional foods and/or pharmaceutical applications. Additionally, incorporating sensory analyses and consumer acceptance studies will be crucial to ensure that these new products meet market expectations and consumer preferences. Concurrently, researching the accumulation of heavy metals by these plants in polluted areas is vital for understanding their potential in phytoremediation, evaluating environmental health risks, and ensuring their safety for consumption.

## Figures and Tables

**Table 1:** Species and subspecies of the *Diplotaxis* genus (WFO Plant list, www.wfoplantlist.org, accessed on 25 April 2024) and their geographical areas (Plants of the World Online, www.plantsoftheworldonline.org, accessed on 25 April 2024) [6,7].

Species (and Subspecies)	Native Geographical Area
*D. acris* (Forssk.) Boiss.	Egypt, Near East (to Iraq)
*D. antoniensis* Rustan	Cape Verde
*D. assurgens* (Delile) Gren. ex Thell.	Morocco
subsp. *tetragona* (Maire) Nègre.	Morocco
*D. berthautii* Braun-Blanq. & Maire	Morocco
*D. brachycarpa* Godron	Northern Algeria
*D. brevisiliqua* (Coss.) Mart.-Laborde	Northwest Africa
*D. catholica* (L.) DC.	Portugal, Spain, Morocco
*D. cretacea* Kotov	Northeast of Ukraine, South of Russia
*D. cyrenaica* (E.A. Durand & Barratte) Maire & Weiller	Moldova, Ukraine, Russia
*D. duveyrierana* Coss.	Libya
*D. erucoides* (L.) DC.	Europe, Northern Africa, Near East (to Iraq)
subsp. *cossoniana* (Reut. ex Boiss.) Mart.-Laborde	Algeria
subsp. *longisiliqua* (Coss.) Gómez-Campo	Morocco and Algeria
*D. glauca* (J.A. Schmidt) O.E. Schulz	Cape Verde
*D. gorgadensis* Rustan	Cape Verde
*D. gracilis* (Webb) O.E. Schulz	Cape Verde
*D. griffithii* (Hook.f. & W. Thomps.) Boiss.	Afghanistan, Pakistan
*D. harra* (Forssk.) Boiss.	Spain, Sicilia, Northern Africa, Near East
subsp. *crassifolia* (Raf.) Maire	Spain, Northern Africa
subsp. *glauca* (J.A. Schmidt) Sobr.-Vesp.	Northern Africa
subsp. *hirta* (A. Chev.) Sobr.-Vesp.	Northern Africa
*D. hirta* (A. Chev.) Rustan & Borgen	Cape Verde
*D. ibicensis* (Pau) Gómez-Campo	East Coast of Spain, Balearic Islands
*D. ilorcitana* (Sennen) Aedo, Mart.-Laborde & Munõz Garm.	East Spain
*D. kohlaanensis* A.G. Miller & J. Nyberg	Northern Yemen
*D. muralis* (L.) DC.	Europe, Northern Africa, Southwest Australia
subsp. *ceratophylla* (Batt.) Mart.-Laborde	Northeast Algeria, Northern Tunisia
*D. nepalensis* H. Hara	Western Nepal
*D. ollivieri* Maire	Southern Morocco
*D. pitardiana* Maire	Northwestern Africa
*D. saharensis (Coss.)* Mart.-Laborde	Northwestern Africa
*D. schweinfurthii* O.E. Schulz	Egypt
*D. siettiana* Maire	Spain, Northern Africa
*D. siifolia* Kunze	Portugal, Spain, Northern Africa
subsp. *bipinnatifida* (Coss.) Mart.-Laborde	Southern Morocco
subsp. *vicentina* (Sampaio) Mart.-Laborde	Southwestern Portugal
*D. simplex* (Viv.) Spreng.	Northern Africa
*D. sundingii* Rustan	Cape Verde
*D. tenuifolia* (L.) DC.	Europe, North of Africa, Near East
*D. tenuisiliqua* Delile	Morocco and Algeria
subsp. *rupestris* (J. Ball) Mart.-Laborde	Morocco
*D. varia* Rustan	Cape Verde
*D. villosa* Boulos & Jall.	Jordan
*D. viminea* (L.) DC.	Europe, Northern Africa, Near East
*D. virgata* (Cav.) DC.	Portugal, Spain, Northern Africa
subsp. *brachycarpa* (Godr.) Nègre	Northwestern Africa
subsp. *cavanillesiana* (Nègre) Maire & Weiller	Portugal, Spain
subsp. *cyrenaica* (Durand & Barratte) Nègre	Northern Africa
*D. vogelli* (Webb) Cout.	Cape Verde
*D. wirtgenii*	Europe

**Table 2:** Nutritional composition of the raw matter of *D. tenuifolia* leaves, *D. erucoides* leaves, and *D. simplex* leaves and flowers [8,14,15,16].

Composition	*D. tenuifolia* Leaves	*D. erucoides* Leaves	*D. simplex* Leaves	*D. simplex* Flowers
Moisture (g 100 g^−1^)	91.00	88.27	69.26	67.21
Ash (g 100 g^−1^)	1.30	2.18	8.28	3.65
Potassium (mg 100 g^−1^)	468.00	157.70	1161.97	1209.95
Calcium (mg 100 g^−1^)	309.00	60.00	359.66	295.11
Magnesium (mg 100 g^−1^)	-	114.10	98.37	167.23
Phosphorus (mg 100 g^−1^)	41.00	47.70	-	-
Sodium (mg 100 g^−1^)	-	14.80	30.74	252.48
Ion (mg 100 g^−1^)	5.20	1.20	19.71	0.52
Zinc (mg 100 g^−1^)	-	0,50	-	-
Copper (mg 100 g^−1^)	-	0.10	0.15	0.16
Protein (g 100 g^−1^)	2.60	2.25	7.03	8.70
Lipids (g 100 g^−1^)	0.30	0.25	0.69	1.38
Fiber (g 100 g^−1^)	0.90	2.93	-	-
Carbohydrate including fiber (g 100 g^−1^)	4.80	7.06	14.75	19.06
Energy (kcal 100 g^−1^)	28.70	27.73	-	-

**Table 3:** Lipid composition of *D. simplex* leaves and flowers, *D. viragata* flowers, and *D. erucoides* non-flowering aerial parts expressed as relative percentages [15,19].

Composition	*D. simplex* Leaves	*D. simplex* Flowers	*D. viragata* Flowers	*D. erucoides* Non-Flowering Aerial Parts
Caprylic acid (C8:0)	-	1.0	-	-
Capric acid (C10:0)	-	0.9	-	-
Lauric acid (C12:0)	-	1.0	-	-
Myristic acid (C14:0)	-	3.4	-	-
Palmitic acid (C16:0)	13.2	15.3	18.2	14.4
Margaric acid (C17:0)	-	0.5	-	-
Stearic acid (C18:0)	-	-	3.1	-
Oleic acid (C18:1)	7.7	6.1	3.4	-
Linoleic acid (C18:2 *n* − 6)	4.4	5.6	23.7	29.1
Linolelaidic acid (C18:2 *n* − 6)	-	-	15.7	6.9
*α*-Linolenic acid (C18:3 *n* − 3)	25.4	27.7	-	-
Arachidic acid (C20:0)	-	2.5	-	-
Ethyl linoleate	14.4	1.4	-	-
Octane	-	2.6	-	-
Nonane	2.8	1.0	-	-
Pentacosane	-	5.1	-	-
Hexacosane	-	5.7	-	-
Phytol	17.6	3.7	-	-
Total	85.5	83.5	64.0	50.4

## Data Availability

Data is contained within the article.
